# Conflict between background matching and social signalling in a colour-changing freshwater fish

**DOI:** 10.1098/rsos.160040

**Published:** 2016-06-01

**Authors:** Jennifer L. Kelley, Gwendolen M. Rodgers, Lesley J. Morrell

**Affiliations:** 1 Centre for Evolutionary Biology and Neuroecology Group, School of Animal Biology (M092), The University of Western Australia, Crawley, Perth, Western Australia 6009, Australia; 2 Genetics, Ecology and Evolution Group, Institute of Integrative and Comparative Biology, University of Leeds, Miall Building, Leeds LS2 9JT, UK; 3 School of Biological, Biomedical and Environmental Sciences, University of Hull, Kingston upon Hull HU6 7RX, UK

**Keywords:** predation, signal conflict, visual communication, camouflage, aggression

## Abstract

The ability to change coloration allows animals to modify their patterning to suit a specific function. Many freshwater fishes, for example, can appear cryptic by altering the dispersion of melanin pigment in the skin to match the visual background. However, melanin-based pigments are also used to signal dominance among competing males; thus colour change for background matching may conflict with colour change for social status signalling. We used a colour-changing freshwater fish to investigate whether colour change for background matching influenced aggressive interactions between rival males. Subordinate males that had recently darkened their skin for background matching received heightened aggression from dominant males, relative to males whose coloration had not changed. We then determined whether the social status of a rival male, the focal male's previous social status, and his previous skin coloration, affected a male's ability to change colour for background matching. Social status influenced skin darkening in the first social encounter, with dominant males darkening more than subordinate males, but there was no effect of social status on colour change in the second social encounter. We also found that the extent of skin colour change (by both dominant and subordinate males) was dependent on previous skin coloration, with dark males displaying a smaller change in coloration than pale males. Our findings suggest that skin darkening for background matching imposes a significant social cost on subordinate males in terms of increased aggression. We also suggest that the use of melanin-based signals during social encounters can impede subsequent changes in skin coloration for other functions, such as skin darkening for background matching.

## 1. Introduction

The colours and patterns of animals facilitate a number of important physiological and behavioural functions, including thermoregulation, mate attraction, rival deterrence and predator avoidance [1]. However, displaying coloration for these purposes can be problematic when the same colours and patterns must serve multiple functions at the same point in time [2,3]. Some taxa, notably reptiles, amphibians, crustaceans, fishes and cephalopods, solve this problem by changing their body coloration to suit the task at hand. For example, colour change in chameleons has evolved as a transient form of communication, serving to facilitate social interactions with conspecifics while allowing them to otherwise remain cryptic to predators [3]. In cephalopods such as octopuses, rapid changes in skin patterning allow a close match to the visual characteristics of the background, serving for camouflage while moving through complex habitats [4]. However, if the same colour pattern is used for multiple functions, then colour change that is beneficial in one context may be detrimental in another.

Conflicting functions are predicted to be particularly problematic for animals that change colour relatively slowly, such as those that display morphological colour changes over a period of days to weeks, as opposed to physiological colour changes that occur rapidly over a few seconds or several hours [5]. Morphological colour change is achieved by changing the density of melanophores (cells that contain melanin pigments) or by altering the amount of melanin pigment within these cells [5,6]. This type of colour change is typically associated with background matching (also referred to as ‘background adaptation’ [5]); animals placed on dark backgrounds disperse their melanin and become darker in coloration while those placed on pale backgrounds aggregate their melanin and appear paler [5]. Individuals that change their coloration to match their visual background, such as fishes, are less likely to be attacked by predators, demonstrating that these colour changes function for camouflage [7,8]. However, animals that change colour relatively slowly may be at increased risk of predation if they move into a new visual environment in which their coloration no longer serves for concealment. A further problem arises if the same coloration is also used as a social signal; in that case changes in skin pigmentation that serve for background matching could disrupt colour changes that function for social signalling.

The melanin patches of coloration often seen in birds and fishes are commonly used to signal social status among rival males [9–11]. In fishes with hierarchical social systems such as salmonids, subordinate males darken their skin coloration, which acts as a signal of subordination to reduce agonistic attacks from dominant individuals [12,13]. There is some evidence that the visual background may also influence these social relationships; juvenile arctic charr (*Salvelinus alpinus*) behave more aggressively on white backgrounds than black backgrounds, but aggression levels diminish rapidly as subordinates increase their melanin pigmentation to signal subordinance [14]. Thus, in fishes, the dual role of changes in melanin pigmentation, both for facilitating background matching and mediating social interactions, provides an excellent opportunity to examine functional trade-offs in adaptive coloration.

In the work reported in this paper, we conducted two experiments aimed at investigating the potential conflict between colour change for background matching and colour change for social signalling. The subject of our experiments was the western rainbowfish (*Melanotaenia australis*), a freshwater fish that shows morphological changes in skin coloration according to its background environment [15–17]. In the first experiment, we determined the relationship between male dominance status and the frequency of aggressive interactions in triads of two males and one female. We then stimulated colour change for background matching in subordinate males (the primary recipients of dominant males' aggression) by placing them on a dark background for a period of one week. The social behaviour of the triad was re-evaluated to establish whether skin darkening for background matching by the subordinate male influenced the level of aggression received from the dominant male. In a second experiment, we determined whether the social status of a rival male inhibits or facilitates skin darkening for background matching in males. We enforced colour change for background matching by placing all fish on a dark background and evaluated the level of skin darkening when the same male was paired with a dominant rival male and with a subordinate rival male. As previous experience can affect interactions between rival males [18], we also considered the effect of order (i.e. whether males were dominant or subordinate in their first social encounter) and previous skin coloration (i.e. before the fish were placed on a dark background) on colour change. If skin coloration serves multiple and conflicting functions, then we expected skin darkening for background matching to be dependent on the rival male's social status.

## 2. Methods

### 2.1. Study species and maintenance

The western rainbowfish (*Melanotaenia australis*) belongs to the family Melanotaenidae, which comprises around 36 species of colourful freshwater fishes that are endemic to Australia and Papua New Guinea [19]. The body coloration of the species is characterized by a series of bright red/orange lateral stripes and a red/orange operculum spot [20]. Previous work has found that males will establish a dominance hierarchy on the basis of body size, when placed in small social groups with large males typically becoming dominant [21,22]. Large males are more likely to outcompete rivals, are preferred by females, and sire a higher proportion of offspring than subordinate males [21,22]. Dominant males also display a high frequency of aggressive behaviours such as fin displays (spreading and flicking the fins) and body shakes and frequently chase subordinate males, excluding them from access to the female [22].

Adult rainbowfish were captured from pools in the upper catchment of the Fortescue River in the Pilbara region of Western Australia in January 2011 (WGS84 coordinates: −21.298, 118.324). Rainbowfish at this site are exposed to predation from fishes such as spangled perch (*Leiopotherapon unicolor*) and Fortescue grunters (*Leiopotherapon aheneus*) [20]. Fish were transported to the Biological Sciences Animal Unit at the University of Western Australia by air. Fish populations were maintained in large mixed-sex groups (group size = approx. 30 fish per tank) in stock tanks (85 × 45 cm, and filled to a water depth of 30 cm) with similar visual backgrounds (brown gravel, an air filter and artificial plants) before trials began. All aquaria were maintained at 26 ± 1°C under identical lighting conditions with a light cycle of 12 : 12 h light : dark.

### 2.2. Experiment 1: does colour change for background matching by subordinate males affect male–male aggressive interactions?

For each experimental group, one female rainbowfish and two males of dissimilar size (all unfamiliar with one another) were selected from stock aquaria. The mismatched males facilitated individual identification without the need for marking and reduced the high levels of aggression that are often associated with closely size-matched males [23]. Each group of three fish was housed in a tank (25 × 30 cm with 12 cm water depth) with brown walls, brown gravel substrate, a plastic plant, an air stone and a transparent front wall and lid, similar to the conditions in the stock tanks. The brown walls were used to provide a homogeneous and standardized visual background. A total of 42 groups (each containing three fish; total number of subjects = 126) were used in the experiment.

The following morning, behavioural observations were conducted to determine the social status of the two males, with the prediction that larger males would be dominant while smaller males would be subordinate [22]. First, a 2 min acclimation period was allowed for the fish to become used to the observer's presence. For a period of 10 min, we recorded the behavioural interactions between the two males and the behaviour of each male towards the females. The behaviours that were recorded included bites (biting or nipping the body), chases (pursuing the male or female around the tank), fin displays (opening the dorsal, pelvic and pectoral fins) and head shakes (rapid sideways head movements) [22]. Courtship and aggressive behaviours are difficult to distinguish in this species, and males are often highly aggressive towards females; for example, fin displays and head shakes are used both in the context of aggression and courtship [21,22]. In all trials, the social status of the two males was unambiguous; dominant males were aggressive towards the other male, causing him to retreat to the corner of the tank, and dominant males directed high levels of courtship activity towards the female.

Following these initial observations, all fish were then removed to individual ‘background treatment’ tanks (18 × 11 cm with 12 cm water depth) for a period of one week. All females (*n *= 42) and dominant males (*n *= 42) were housed in brown tanks (brown walls and gravel) that mimicked the stock conditions, and the tanks in which the behavioural assays were performed (described above). Half of the subordinate males (*n *= 21) were housed in identical conditions to the females and dominant males (i.e. brown tanks, ‘control’), whereas the other half formed the ‘dark’ treatment group, and were housed in tanks with black walls and gravel, but identical in all other details. In accordance with previous studies with this species [15,16], we expected fish to darken their skin coloration for background matching in the dark habitat treatment, but to show no change in skin coloration in the control tanks. After a period of one week in the background treatment tanks, fish were returned to their original social groups (comprising the same individuals) and observation tanks. As above, following 2 min of acclimation to the observer, the social interactions among males and females were observed in each of the 42 tanks.

#### 2.2.1. Statistical methods

All analysis was carried out in R v. 3.2.3 ‘wooden Christmas tree’ [24]. We first compared the overall frequency of behaviours performed by males towards rival males and by males towards females, allowing us to identify which males (dominant or subordinate) performed most of the behaviours observed. Only dominant males performed bites (*n *= 20 males) and headshakes (*n *= 4) towards subordinates; these behaviours were never directed from subordinates to dominants. Dominant males also performed the majority of bites (*n *= 17) and headshakes (*n *= 9) towards females (compared with *n *= 1 for subordinates for each behaviour). Thus, these behaviours were excluded from our subsequent statistical analyses. Generalized linear mixed effects models with Poisson error distributions (as appropriate for count data) were used to test the effect of male social status (dominant/subordinate) on the frequency of chases, fin displays and the total frequency of aggressive behaviours observed before the colour treatment. Group identity was entered as a random factor to control for the repeated measures nature of the data, and an observation-level random effect was added to account for overdispersion [25]. We next investigated whether there were differences in male–male aggression after the background treatment (subordinate males exposed to dark background or control) for dominants and subordinates separately using a generalized linear mixed effects model with a Poisson error distribution. We included the total number of aggressive acts performed by that male as a covariate to control for any individual differences in aggression. Again, we included group identity as a random factor, and an observation-level random effect to account for overdispersion. Non-significant interactions were removed, and only the model with main effects is presented here.

#### 2.2.2. Results

**Figure 1.**
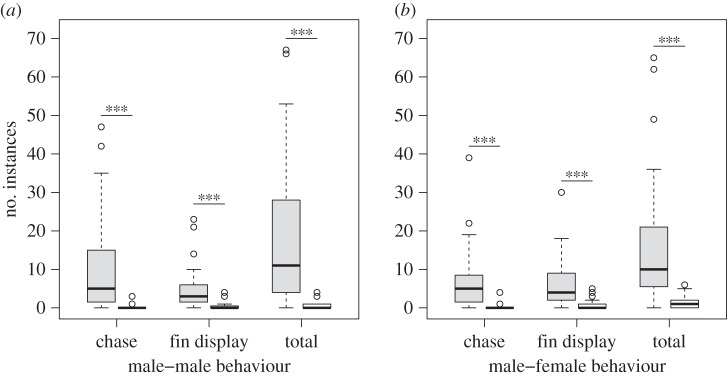
Boxplots show number of chases, fin displays and the total number of aggressive behaviours directed by (*a*) dominant males towards subordinate males (grey bars) and by subordinate males to dominant males (white bars) during the 10 min observation period. (*b*) The frequency of these behaviours performed by males (dominant, grey bars; subordinate, white bars) towards females. Asterisks denote significant differences in the frequency of behaviours performed by dominant and subordinate males (***p < 0.001).

Dominant males performed the majority of behaviours observed (figure 1*a*) and directed more chases and fin displays towards subordinates than subordinates did towards dominant males (table 1*a*). Indeed, subordinates were rarely observed directing aggression towards dominant males (figure 1). Dominant males also directed more behaviour towards females, displaying a higher frequency of chases and fin displays than subordinate males (table 1*b* and figure 1*b*). Males assigned as dominant tended to be larger than those assigned as subordinate (dominants: mean SL ± s.e. = 53.2 ± 1.1 mm; subordinates: 47.2 ± 1.1 mm; *t*_38_ = 6.11, *p* < 0.001).

**Table 1.** Generalized linear mixed effects models testing for the effect of social status (dominant or subordinate) on the frequency of behaviours males directed towards the other male (*a*) and towards the female (*b*) before the colour change treatment. Significant *p*-values are highlighted in italics.

behaviour	estimate	s.e.	*Z*-value	*p*-value
(*a*) male–male interactions				
chase	−4.353	0.538	−8.088	*<0*.*001*
fin display	−2.541	0.340	−7.472	*<0*.*001*
total	−3.572	0.358	−9.978	*<0*.*001*
(*b*) male–female interactions				
chase	−3.519	0.424	−7.704	*<0*.*001*
fin display	−1.907	0.247	−7.709	*<0*.*001*
total	−2.543	0.266	−9.565	*<0*.*001*

**Figure 2.**
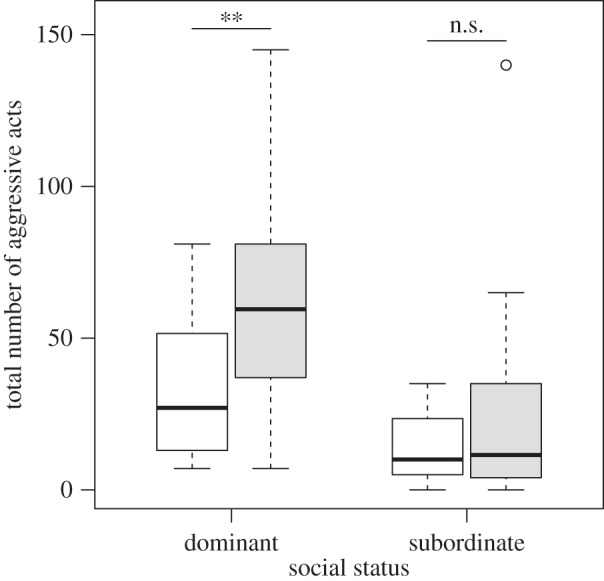
Boxplot displays the total frequency of aggressive acts performed over a 10 min observation period by dominant and subordinate males after subordinate males had experienced either a dark background (grey bars) or a control background (white bars). Asterisks denote significant differences in male behaviours (**p < 0.01).

After the colour treatment, there was a significant effect of treatment (dark background or control) on the total number of aggressive acts performed by dominant (table 2*a*) but not subordinate (table 2*b*) males (figure 2). Thus, dominant males were more aggressive towards subordinates that had experienced dark backgrounds than those that had experienced control backgrounds, whereas subordinate behaviour was not dependent on the background they had experienced. As in our initial observations, dominant males thus remained more aggressive overall than subordinates (figure 2). Previous aggressive behaviour had no significant effect on subsequent behaviour, and did not interact with colour treatment (table 2).

**Table 2.** Results of the generalized linear mixed effects models investigating the effect of colour change treatment (dark background or control) and previous aggressive behaviour on the total frequency of aggressive behaviours performed by dominant (*a*) and subordinate (*b*) males. Significant *p*-values are highlighted in italics.

	estimate	s.e.	*Z*	*p*-value
(*a*) dominant males				
intercept (treatment)	4.344	0.218		
colour (dark treatment)	0.664	0.245	2.708	*0*.*0067*
previous behaviour	−0.011	0.007	−1.564	0.1179
(*b*) subordinate males				
intercept (control)	1.885	0.390		
colour (dark treatment)	−0.457	0.489	0.935	0.350
previous behaviour	0.228	0.207	1.102	0.271

### 2.2. Experiment 2: does the presence of a rival male and previous social experience affect colour change for background matching?

The second experiment investigated whether the social status of a rival male influenced colour change for background matching when both males (and the female) were placed on a dark background. As a limited number of fish was available for these experiments, we re-used fish from the first experiment, but only those males that had not experienced a change in skin coloration (i.e. all dominant males and subordinate males from control backgrounds only). We selected 30 males of intermediate body length that were assigned as new focal fish (*n* = 30), whereas males that were larger or smaller than focal males were designated as stimulus fish (*n* = 30). We also used females that had taken part in the previous experiments to provide a stimulus for male–male interactions. We ensured that none of the individuals selected (males or females) had interacted together previously.

Focal males were exposed to larger and smaller stimulus males in succession, allowing the same focal fish the experience of being both dominant (the stimulus fish was smaller in size) and subordinate (the stimulus fish was larger in size). Half of the test fish experienced a smaller stimulus male first, whereas the other half were exposed to a larger stimulus male first. Before fish were assigned to their treatment tanks, the left side of each fish was then photographed (out of water) with a digital SLR camera (Nikon D90) using a fixed aperture (f/11) and variable shutter speed (1/400–1/600 s). Fish were illuminated under broad-spectrum lighting (2 × 11 W fluorescent bulbs) and a colour standard (Munsell ColorChecker™) was included in every image. No anaesthetic was used to photograph the fish, because the procedure only took a few seconds, and anaesthesia is known to affect coloration in teleost fishes [26].

Each pair of males was housed with a single female, and each group interacted on a dark background (described in experiment 1) to stimulate colour change for background matching over a period of 3 days. We reduced the time period of colour change to 3 days (cf. one week in the first experiment) both to reduce stress to the fish (through increased aggression) and because we noted that 3 days was sufficient to stimulate changes in melanin pigmentation [27]. We further prevented injury to fish by preventing triads from interacting physically by dividing the treatment tank into three sections with perforated transparent walls, such that visual and olfactory cues were available. Females were positioned in a larger compartment at the back of the tank while the males were located adjacent to one another at the front (to facilitate monitoring), and were therefore exposed to equal areas of the visual background from the surrounding sidewalls. Although we did not record the behaviour of the males and females in this part of the experiment, the formation of social hierarchies during the 3 day period was consistent with our previous observation, i.e. dominant males displayed high levels of aggression.

Following the 3 day colour change period, males were re-photographed, and each social group was placed in a brown holding tank (conditions as for experiment 1) for a period of one week, so that fish reverted back to their original coloration (i.e. reduced their melanin pigmentation). Fish were photographed again, prior to being placed on the dark background, so that their coloration prior to their second social experience could be evaluated. Focal males that had previously experienced a larger stimulus male (i.e. were subordinate) were then paired with a smaller stimulus male (i.e. were now dominant) and vice versa, ensuring that all fish were unfamiliar with one another. All male pairs thus interacted with a new female and were housed on a dark background for a further period of 3 days. At this end of this second social encounter, fish were photographed, before being returned to mixed-sex stock tanks, where they played no further part in the experiment. Fish in experiment 2 were therefore photographed four times in total (before and after each social encounter).

All of the digital images (saved as TIFF files) were analysed using ImageJ software, v. 1.44 (http://rsb.info.nih.gov/ij/index.html). Images were first standardized for size using a spatial scale included with each photograph, and the body length of each fish was measured in millimetres. We assessed the proportion of melanin pigmentation present on the body of male fish by adapting our previous methods for measuring overall skin darkness [15]. Using ImageJ, each TIFF image was first standardized for brightness using the greyscales on the Munsell™ colour standard before converting the image to greyscale (eight-bit). The outline image of each fish's body was then traced to measure the overall body area, excluding the fins. We then evaluated overall skin darkness by using the threshold function in ImageJ to calculate the proportion of body area with pixel intensities lower (i.e. darker) than the threshold value of 70. These methods were used for the photographs of each male that were taken prior and subsequent to the evaluations of social status or assignment to background treatment.

#### 2.2.1. Statistical methods

We used a linear model to assess whether there were differences in skin coloration (percentage black body coloration) between males assigned to different statuses before the colour treatment, for both the first and second exposure. Subsequently, we explored the effect of social status (dominant or subordinate), the order in which males experienced each social role (dominant or subordinate first) and their interaction on change in percentage black body coloration. We used a linear mixed effects model with tank identity as a random factor to account for the repeated measures nature of the data. To account for any potential effects of coloration based on the males' previous social role, we included previous coloration as a covariate. Non-significant interactions were removed in a stepwise manner following Crawley [28]. Pairwise comparisons within the main dataset, controlling for body size, were made using linear (ANCOVA) and linear mixed effects models (repeated measures data) as appropriate. Normality of residuals was assessed using visual inspection of diagnostic plots, accompanied by Shapiro–Wilks tests. All indicated that the model assumptions were met.

#### 2.2.2. Results

Prior to being allocated to the dark background to induce colour change for background matching, there was no effect of social status (for the first trial) on the percentage of black body coloration (LM: *t* = 0.154, d.f. = 1,28, *p* = 0.787). This indicates that all males were of a similar colour before being placed into the experimental tanks to experience either dominant or subordinate status in the first exposure. For the second exposure, however, males that were to experience subordinate status (and had therefore previously been dominant) were darker than those that were to experience dominance (LM: *t* = −2.513, d.f. = 1,28, *p* = 0.018).

**Figure 3.**
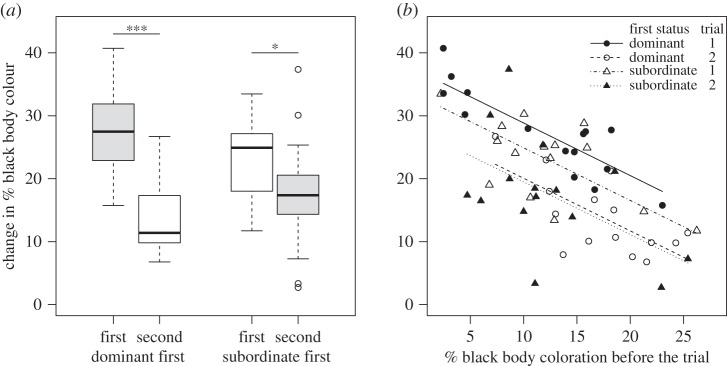
Boxplots (*a*) illustrate the effect of social status on the extent of colour change (skin darkening) for background matching. Dark bars are colour change in the first trial, light bars are colour change in the second trial, for fish that experienced dominance first (left), and those that experienced subordinate status first (right). Each focal fish experienced being both dominant and subordinate. (*b*) The effect of previous body coloration on the percentage change in body coloration for fish that were dominant first (circles) or subordinate first (triangles), when they held dominant status (filled symbols) or subordinate status (open symbols). Lines represent the predictions of the linear mixed effects model reported in table 3.

**Figure 4.**
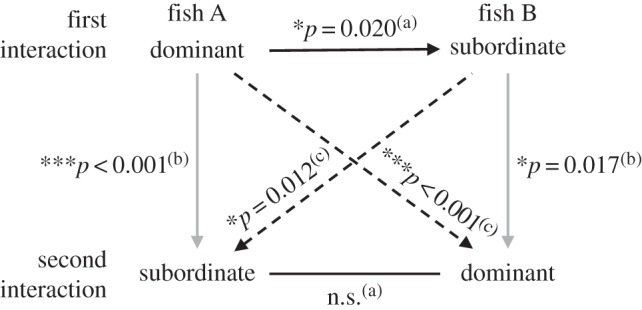
Illustration of the effect of male social status (dominant or subordinate), exposure (first or second trial) and their interactions on colour change (skin darkening). Solid grey lines depict the same individual observed in different social roles, dashed lines are different fish observed in the same social role, whereas solid black lines are comparisons of dominant and subordinate fish within a trial. Arrows point from the fish that darkened most to the ones that darkened least. Superscript letters link to the statistical comparisons shown in table 4 (*p < 0.05; **p < 0.01; ***p < 0.001).

There was a significant interaction between social status and the order in which the statuses were experienced on percentage change in black body coloration, and a significant independent effect of previous coloration (table 3; figures 3 and 4). For both dominant and subordinate males, darker males showed a smaller change in body coloration when exposed to a dark background than paler males (table 3 and figure 3). All subsequent tests thus included previous coloration as a covariate (which was significant in all cases; table 4). There was a significant difference in colour change between dominant and subordinate males in the first interaction, with dominant males darkening more than subordinates, but there was no difference in colour change according to social status for the second interaction (table 4*a*; figures 3 and 4). When considering colour change of the same individual in both social roles, fish darkened more in their first interaction (irrespective of social status) than they did in their second interaction (table 4*b*; figures 3 and 4). When examining fish in the same social role over different interactions, males that experienced dominance first darkened more than those that experienced dominance second, and when subordinate, males that experienced being subordinate first darkened more than those that were subordinate second (table 4*c*; figures 3 and 4).

**Table 3.** Results of linear mixed effects model on the effect of social status, and the order that males experienced each status, and previous coloration on the extent of colour change for background matching. Significant *p*-values are highlighted in italics. The intercept represents colour change in dominant males that were dominant in their first interaction.

	value	s.e.	d.f.	*t*	*p*-value
intercept	37.204	2.202			** **
social status	−9.362	2.004	28	−4.672	*<0*.*001*
order experienced	−8.785	1.822	27	−4.820	*<0*.*001*
previous coloration	−0.835	0.121	27	−6.901	*<0*.*001*
social status × order	14.198	2.493	27	5.695	*<0*.*001*

**Table 4.** Pairwise comparisons of male colour change (skin darkening) compared across social roles (dominant or subordinate) and exposures (first trial or second trial), controlling for percentage black body coloration (‘colour’) before the trial. ‘Contrast’ refers to the comparison described in the section heading. In each case, the intercept refers to the trial and status in the first column, and the contrast is made with that in the second column. Significant *p*-values are highlighted in italics.

trial and status	trial and status	fixed effect	estimate	s.e.	d.f.	*t*-value	*p*-value
(*a*) *comparison of dominant and subordinate colour change within a trial (ANCOVA)*							
1: dominant	1: subordinate	intercept	36.879	1.919			
		contrast	−3.959	1.598	1,27	−2.477	*0*.*020*
		colour	−0.808	0.130	1,27	−6.194	*<0*.*001*
2: dominant	2: subordinate	intercept	27.576	3.222			
		contrast	0.468	2.626	1,27	0.178	0.860
		colour	−0.814	0.224	1,27	−3.636	*0*.*001*
(*b*) *comparison of colour change for individual fish (A or B) in each social role (linear mixed effects)*							
1: dominant	2: subordinate	intercept	37.672	2.453			
		contrast	−8.571	1.567	13	−5.471	*<0*.*001*
		colour	−0.875	0.128	13	−6.822	*<0*.*001*
1: subordinate	2: dominant	intercept	27.295	3.095			* *
		contrast	−5.126	1.980	13	−2.735	*0*.*017*
		colour	−0.791	0.209	13	−3.788	*0*.*002*
(*c*) *difference in colour change by fish in dominant and subordinate roles (ANCOVA)*							
1: dominant	2: dominant	intercept	37.504	2.683			
		contrast	−9.352	2.236	1,27	−4.183	*<0*.*001*
		colour	−0.861	0.182	1,27	−4.720	*<0*.*001*
1: subordinate	2: subordinate	intercept	32.128	2.407			
		contrast	−5.304	1.969	1,27	−2.694	*0*.*012*
		colour	−0.743	0.168	1,27	−4.433	*<0*.*001*

## 3. Discussion

Here we reveal that changes in body coloration that are associated with skin darkening for background matching also mediate aggressive interactions between rival males. Specifically, we found that subordinate males that had recently darkened their coloration received more attacks from dominant conspecifics than control males. We also showed that when pairs of rival males were placed on a darkened background to enforce skin colour change, the extent of skin darkening was dependent on a male's previous social experience, his current social status and his previous body coloration. Specifically, dominants showed stronger skin darkening than subordinate males in their first social encounter, but social status did not influence skin darkening in the second encounter. Skin colour change was also affected by previous coloration, with darker males showing a smaller change in body coloration when placed on a dark background than paler males. While the effect of social experience on the outcome of aggressive encounters (so-called winner or loser effects) is well known in animals such as birds, reptiles and fishes [18], our results suggest that previous social encounters, and the change in body coloration that results from these interactions, can determine subsequent colour change for background matching. Our findings reiterate the multiple roles of animal coloration and suggest that past social relationships can impact on other key roles of skin coloration, such as camouflage.

The findings from the first experiment revealed that recently darkened males were subjected to high levels of aggression from dominant males, suggesting that there is a high social cost of skin darkening for background matching in subordinate males. This effect is due to a change in the behaviour of dominant males rather than subordinates, because darkened subordinate males did not initiate more attacks towards dominant males as a result of their treatment. Melanin is often used to signal social status in animals such as birds [29–31], fishes (reviewed by [10,32]) and insects [33]. If skin darkening is used to signal dominance in western rainbowfish, then darkened subordinate males may have experienced heightened aggression from dominant males because they were falsely displaying a signal of social status. Similar findings have been reported in birds; in house sparrows (*Passer domesticus*) for example, the size of the black chest bib positively correlates with male dominance [34–37]. Artificially enhancing the size of the dominance signal in subordinate house sparrows incites increased aggression from dominant birds, suggesting that incorrectly signalling social status is costly [37]. Similarly, artificial manipulation of the black facial markings of female paper wasps (*Polistes dominulus*), which are used to signal social dominance, results in a significant increase in aggression directed towards dishonest signallers than towards experimental controls [33].

Our second experiment revealed that recent social experience (less than one week), and the order of social status experienced plays a role in determining current colour change for background matching. In the first social encounter, dominant males darkened more than subordinate males when placed on a dark background. Subordinate males may have darkened less because of the potential social costs associated with darkening and inciting increased aggression from dominant males (as in experiment 1). One previous study has examined whether colour change for social signalling depends on the visual background. In this study, subordinate male arctic charr that were placed on white backgrounds increased their melanin pigmentation to signal their subordinate social status instead of lightening their skin to match the pale background [14]. Collectively, these studies suggest that subordinate males are compelled to maintain their social signals [14], or are potentially inhibited from falsely displaying these signals (this study) when placed on a background that would otherwise induce a change in skin colour. That males are prepared to potentially increase their vulnerability to visual predators by not changing colour to match their visual background suggests that in animals that change colour relatively slowly (over several days), the benefits of social signalling can outweigh the potential costs associated with increased conspicuousness.

We did not observe an effect of social status on skin darkening for males taking part in their second social encounter. The observation that all males, irrespective of social status, darkened more in their first social interaction than in the second may be due to the lasting effects of skin darkening from the first social interaction. Prior to the second social encounter, dominant males remained darker than subordinates despite all fish spending one week on a control background. Thus, it appears that a one week period is not sufficient to fully reverse the combined effects of the dark background and social status experience on male skin coloration. Although we accounted for this effect by considering previous coloration in our analyses, this carryover effect may explain why social status did not affect skin colour change during the second social encounter. Furthermore, in all cases, colour change for background matching was influenced by an individual's previous coloration, with darker fish showing less change in coloration than pale fish. This highlights the physiological limits associated with colour change, because further colour change may be impeded when maximum darkening is already achieved (e.g. in dominant males on a dark background) and no further change can occur, because all melanin is dispersed. Repeated exposure to a particular visual environment can also influence melanin pigmentation; for example, zebrafish that experienced a change in background colour every 2 days (alternating between black and white tanks) increased their overall area of melanin pigmentation over successive exposures and developed more rapid responses, which indicates that learning may be involved in colour change [38].

The finding that colour change for background matching was influenced by social status and previous social experience is also consistent with the ‘loser effect’, which is a tendency for an animal that loses a prior contest to also lose in a subsequent contest, in contrast to the ‘winner effect’, which describes the increased probability of winning future fights (reviewed by [39]). Rainbowfish that were subordinate in their first social role (i.e. ‘losers’) did not become darker in their second social role when they were dominant, such that social status did not affect skin darkening in the second social encounter. Winner and loser effects have been described in a number of taxa in the context of male fighting ability [40–42] and are thought to occur because of differences in the willingness of males to engage in, or escalate a contest [39]. Although we are not aware of any studies investigating the role of previous social experience in the context of colour change for background matching, studies with lizards have revealed that previous social experience can often be a more important determinant of contest outcome than asymmetries in signalling traits such as coloration and body size [43,44]. The evidence presented here suggests that winner/loser effects could also impact on the use of coloration for other functions, such as camouflage.

We did not perform a reciprocal treatment where subordinate males were placed on a pale background (to induce skin lightening) because we were particularly interested in understanding the relationship between skin darkening and aggression, which is relatively well known in fishes [12,14,45]. It is therefore unclear whether skin lightening by subordinate fish would incite or inhibit aggression from the dominant male, or have no effect on aggressive behaviour. Furthermore, it is possible that skin darkening by the treatment males may have affected some additional components of the social interaction, for example, if skin darkening impedes individual recognition and dominant males increased their aggression to re-establish their social status with unfamiliar males (i.e. those from the dark treatment). However, this is unlikely, because skin changing for background matching would then disrupt other key behaviours that rely on individual recognition (e.g. mate choice, cooperation during predator inspection), and there is evidence that specific components of patterning (e.g. facial markings) are used for individual recognition and occur independently of overall body coloration [46,47].

Changes in melanin pigmentation in fishes are associated with stress responses, because sustained levels of stress stimulate the release of alpha melanocyte stimulating hormone (α-MSH) and adrenocorticotrophic hormone that are associated with skin darkening and aggression in teleost fishes [48]. In subordinate arctic charr, skin darkness is positively correlated with levels of both α-MSH and plasma cortisol suggesting that a low social status (being subjected to aggression) is stressful [45]. However, there is no effect of environmental background colour on plasma α-MSH levels in arctic charr [14]. Melanin pigmentation is correlated with aggressive behaviour in mosquitofish (*Gambusia holbrooki*) which exhibit a temperature-sensitive male polymorphism; melanic males are more aggressive towards females, spending more time chasing and engaging in a greater proportion of (thrusting) mating attempts than silver morphs [49,50]. On the other hand, melanic individuals may benefit through experiencing reduced physiological and behavioural responses to stress; for example, ‘spotted’ Atlantic salmon (*Salmo salar*) with highly pigmented skin had a lower cortisol response to stress and showed reduced locomotory behaviour than ‘non-spotted’ salmon [51]. Melanin pigmentation is therefore not only used to fulfil a variety of functional roles in teleosts, but the underlying physiological and endocrine pathways are complex and interacting [9]. Indeed, interactions among the underlying physiological pathways may partly explain trade-offs in the multiple functions of melanin-based pigments.

In summary, examining trade-offs in colour pattern functions in organisms with changeable coloration can provide valuable insights into the selective pressures that shape animal coloration, but studies are surprisingly scarce [3]. The results presented here suggest a direct conflict between the role of melanin in mediating aggressive interactions among rival males and skin darkening for camouflage through background matching. Consequently, individuals must make behavioural decisions that minimize their conspicuousness to predators while maintaining signals of social status to rival conspecifics.

## Data accessibility

Original data are publicly available on the external repository Dryad (http://dx.doi.org/10.5061/dryad.271k9).
